# A powerful qPCR-high resolution melting assay with taqman probe in *plasmodium* species differentiation

**DOI:** 10.1186/s12936-021-03662-w

**Published:** 2021-02-28

**Authors:** Aline Lamien-Meda, Hans-Peter Fuehrer, David Leitsch, Harald Noedl

**Affiliations:** 1 Institute for Specific Prophylaxis and Tropical Medicine, Medical University of Vienna, Vienna, Austria; 2 Institute of Parasitology, University of Veterinary Medicine, Vienna, Austria; 3 Malaria Research Initiative Bandarban, Vienna, Austria

**Keywords:** Malaria, qPCR, HRM, *Plasmodium*, *Plasmodium falciparum*, *Plasmodium ovale wallikeri*, *Plasmodium ovale curtisi*, *Plasmodium vivax*, *Plasmodium knowlesi*, *Plasmodium malariae*

## Abstract

**Background:**

The use of highly sensitive molecular tools in malaria diagnosis is currently largely restricted to research and epidemiological settings, but will ultimately be essential during elimination and potentially eradication. Accurate diagnosis and differentiation down to species levels, including the two *Plasmodium ovale* species and zoonotic variants of the disease, will be important for the understanding of changing epidemiological patterns of the disease.

**Methods:**

A qPCR-high resolution melting (HRM) method was to detect and differentiate all human *Plasmodium* species with one forward and one reverse primer set. The HRM detection method was further refined using a hydrolysis probe to specifically discriminate *Plasmodium falciparum*.

**Results:**

Out of the 113 samples tested with the developed HRM-qPCR- *P. falciparum* probe assay, 96 (85.0 %) single infections, 12 (10.6 %) mixed infections, and 5 (4.4 %) were *Plasmodium* negative. The results were concordant with those of the nested PCR at 98.2 %. The assay limit of detection was varied from 21.47 to 46.43 copies /µl, equivalent to 1–2.11 parasites/µl. All *P. falciparum* infections were confirmed with the associated Taqman probe.

**Conclusions:**

Although the dependence on qPCR currently limits its deployment in resource-limited environments, this assay is highly sensitive and specific, easy to perform and convenient for *Plasmodium* mono-infection and may provide a novel tool for rapid and accurate malaria diagnosis also in epidemiological studies.

## Background

A significant step-up in worldwide malaria control efforts in the past decades has resulted in a considerable reduction of mortality and clinical episodes in many malaria-endemic countries [1]. At the same time, asymptomatic infections have gained importance as a reservoir of new infections and epidemics. Novel and more sensitive tools are, therefore, urgently needed to support when technically possible, microscopic examination of thick and thin bloods films remaining the gold standard for laboratory diagnosis of malaria in resource-limited environments.

In Europe and European Economic Area, 8349 malaria cases were reported in 2018 and nearly all reported cases were imported. Around 84 % of imported malaria cases have been reported to be non-falciparum malaria [2]. These tend to receive limited attention due to their less severe clinical course (when compared to *Plasmodium falciparum*); however, recent and increasing numbers of studies are supporting the capacity of *Plasmodium vivax* to cause severe disease by affecting the spleen, lungs and born marrow [3–7]. These findings are prompting a more thorough and comprehensive differentiation between species for a correct treatment approach, particularly in regions with declining *Plasmodium* endemicity [8].

In spite of a similar presentation in the early stages of the disease *P. vivax* and *Plasmodium ovale* spp. pose specific challenges due to their ability to produce dormant liver stages (hypnozoites). These can induce re-activation of malaria up to several years after the initial infection [9]. Infection with *Plasmodium malariae*, known as quartan fever, can result in long-lasting disease if not well treated. *Plasmodium ovale* spp. and *P. malariae* were reported to be responsible for asymptomatic cases in a seroprevalence study of *P. ovale* spp. and *P. malariae* in healthy populations in Western Africa [10].

The *Plasmodium* mitochondrial genome (6-kb) is rooted entirely from the female gametocyte and does not undergo recombination among lineages. That makes it a desirable candidate for pathogens surveillance and for *Plasmodium* species diagnosis. Additionally, the mitochondrial genome exists in multiple copies (up to ~ 22 copies) defining it as a good target for *Plasmodium* species differentiation [11–16].

Light microscopy and immuno-chromatographic rapid diagnostic tests (RDTs) are the two methods recommended by the World Health Organization (WHO) and used routinely for parasitological diagnosis of malaria [17]. However, particularly in asymptomatic infections and in the case of low parasite densities, misclassification and low detection rates of non-falciparum malaria are commonly reported with microscopy and RDTs, respectively [18]. Multiplex qPCR has been identified as a substantial improvement to microscopy in reference to laboratory detection of malaria species specifically due to its superior limit of detection (LOD) [18].

Since the development of PCR based methods in malaria diagnosis in the late 1980s, several methods targeting the *Plasmodium* 18S SSU RNA gene have been developed. These methods include isothermal amplification (LAMP), conventional nested and semi-nested PCRs, and real-time PCRs [19, 20]. However, *Plasmodium* species-specific identification by these methods requires multiplexing or many time-consuming steps using primer pairs that are specific to each of the *Plasmodium* species.

High-Resolution Melting (HRM) curve analysis is a fast and straightforward post-PCR analysis which has been successfully applied for genotyping, including pathogen-typing. In this procedure, the region of interest is amplified in the presence of a specialized DNA binding dye and a gradual denaturation of the amplicons, which produce characteristic melting profiles. Recently, an HRM assay targeting the 18S SSU RNA was described for simultaneous detection and typing of five *Plasmodium* species affecting humans [21]. The use of such an HRM assay would help saving time in *Plasmodium* species identification. HRM technology was successfully used to differentiate both *P. ovale* species in one PCR reaction by targeting the highly conserved apicoplast genome [22].

In this paper, a qPCR high-resolution assay is described targeting the mitochondrial DNA for simultaneous detection and quantification of *P. falciparum, P. vivax, P. malariae, P. ovale curtisi, P. ovale wallikeri* and *Plasmodium knowlesi*, with high specificity and sensitivity. A Taqman probe was added to the PCR mix to specifically detect and confirm *P. falciparum* infection.

## Methods

### Samples and DNA extraction

Samples (113) from published studies conducted in Bangladesh, Malaysia and Ethiopia were used for method development (Additional file 1). All samples were collected under approved protocols and after obtaining written informed consent. Parasite density and species diagnosis were initially established by microscopy and nested PCR [23, 24]. Archived filter papers of each sample (4 × 4 mm blood spots soaked overnight in 100 µl PBS at 4°C) were used for DNA extraction with Illustra blood genomicPrep Mini Spin kits (GE Healthcare, Buckinghamshire, UK) following the manufacturer’s protocol. The DNA was eluted with two times 50 µl of elution buffer and stored at − 20 °C.

### Target selection and primer design

The mitochondrial (complete or partial) genome of *P. falciparum* (KT119883, KT119882), *P. ovale* spp. (AB354571, HQ712052, HQ712053), *P. vivax* (KF668406, AY598121), *P. malariae* (AB489192, AB354570), and *P. knowlesi* (AY598141, AY722797) obtained from GenBank (National Center for Biotechnology Information, Bethesda, MD) were used for primer design.

**Fig. 1:**
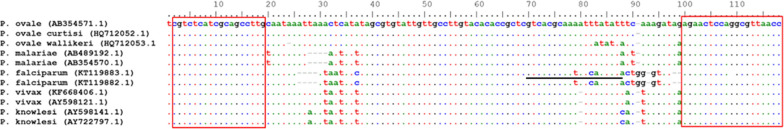
Selected fragment of *Plasmodium* cox1 gene with human *Plasmodium* species signatures: Fragments: *P. falciparum* (110 bp); *P. malariae* (112 bp); *P. ovale wallikeri*, *P. vivax* and *P. knowlesi* (115 bp); *P. ovale curtisi* (116 bp); PhHRM F1 (18 bp); PhHRM R1 (20 bp); PhHRM probe (22 bp: 5’ VIC- ctcgtcacgcaatatcaatata-MBG-NFQ 3’) with the corresponding bp on the fragment (black horizontal line). The used primer base pairs are presented in a red rectangle

The selected fragment of all six *Plasmodium* species is presented in Fig. 1. The partial mitochondrial genome was aligned using the clustalW algorithm, as implemented in the BioEdit software package version 7.2.6. After the identification and selection of a specific and conserved region, a pair of primers, specific to all 6 *Plasmodium* species was designed to amplify a 109–117 bp fragment for the real-time PCR-HRM assay (qPCR-HRM) using the Primer3 online tool. The primers were synthesized by Eurofins MWG Synthesis GmbH (Ebersberg, Germany) and purified by reverse-phase high-performance liquid chromatography.

### PCR and melting curve

The PCR reaction was performed in 20 µl containing 100 nM forward primer (PhHRM F1: 5’-CGTCTCATCGCAGCCTTG- 3’), 100 nM reverse primer (PhHRM R1: 5’-AGGTTAACGCCTGGAGTTCT-3’), 1x GoTaq qPCR master mix (Promega Corporation, Madison, USA), 50 nM of Texas red probe (5’ TR-GTCACGCAATATCAATATA-MGB-Eclipse 3’) (Eurogentec, Liège, Belgium) and 4 µl DNA sample. The PCR was performed in a Roche LightCycler 480 qPCR system (Roche Diagnostics GmbH, Mannheim, Germany) with an initial denaturation step at 95 °C for 3 min, followed by 45 cycles of 95 °C for 10 sec and 62 °C for 30 sec. The PCR products were then subjected to the following melting programme: denaturation at 95°C for 1 min, cooling to 65 °C (held for 1 min), and continuous heating at 2.2°C/s with fluorescence acquisition from 65 °C to 95 °C. Two filter combinations were used: SYBR Green/ HRM dye and 533–610/Texas-red dye.

### Positive control plasmids preparation and sequencing

The short fragment of each *Plasmodium* species (Fig. 1) was inserted into TOPO vector using the TOPO® TA Cloning® Kit, and the recombinant vector was transformed into competent *Escherichia coli*. Selected positive clones were cultured, the plasmids purified and sequenced by Eurofins MWG Synthesis GmbH (Ebersberg, Germany). The sequencing data were analysed using Vector NTI.10 (Invitrogen) software, and the sequences were checked by using the Basic Local Alignment Search Tool (Nucleotide BLAST) to confirm their identity.

### Assay sensitivity, specificity and precision

The method specificity was evaluated with the melting profile (comparatively to the positive control plasmid), and also with DNA from the following organisms (using identical PCR conditions): *Toxoplasma gondii, Leishmania infantum, Trypanosoma brucei, Trypanosoma cruzi, Babesia divergens, Entamoeba histolytica, Cryptosporidium parvum, Giardia intestinalis, Enterocytozoon bieneusi, Encephalitozoon cuniculi, Pneumocystis jirovecii, Echinococcus granulosus, Strongyloides stercoralis, Dirofilaria repens, Toxocara canis, and Ascaris suum.* Five *Plasmodium* negative blood spots on filter papers from human were tested. The assay was performed in duplicate with each DNA sample.

The PCR amplification efficiency was established by the means of three calibration curves providing the mean PCR efficiency and analytical sensitivity. The PCR efficiency was calculated according to the following formula:

PCR efficiency = 10 ^− 1/slope^ − 1 [25].

The assay sensitivity was expressed as the limit of detection (LOD) at 95 % probability. The LOD for each *Plasmodium* species was defined as the measured concentration producing at least 95 % positive replicates [26]. The LOD was assessed by amplifying seven different concentrations (80, 60, 40, 20, 10, 8, and 6 copies/µl) of each plasmid in six (6) replicates on four separate occasions. The total proportion of positive tests was recorded and subjected to probit regression analysis using R version 3.4.2 (2017-09-28) via RStudio version Version 1.1.383 to obtain LOD with confidence interval (CI). Similarly, the boxplots of the melting temperatures (Tm) were also produced using R via the RStudio version. The Welch’s unequal variances t-test was used to compare the difference between arithmetic means of the respective Tm of the amplicons of all five parasite species using R.

## Results

### Assay design and optimization

**Fig. 2:**
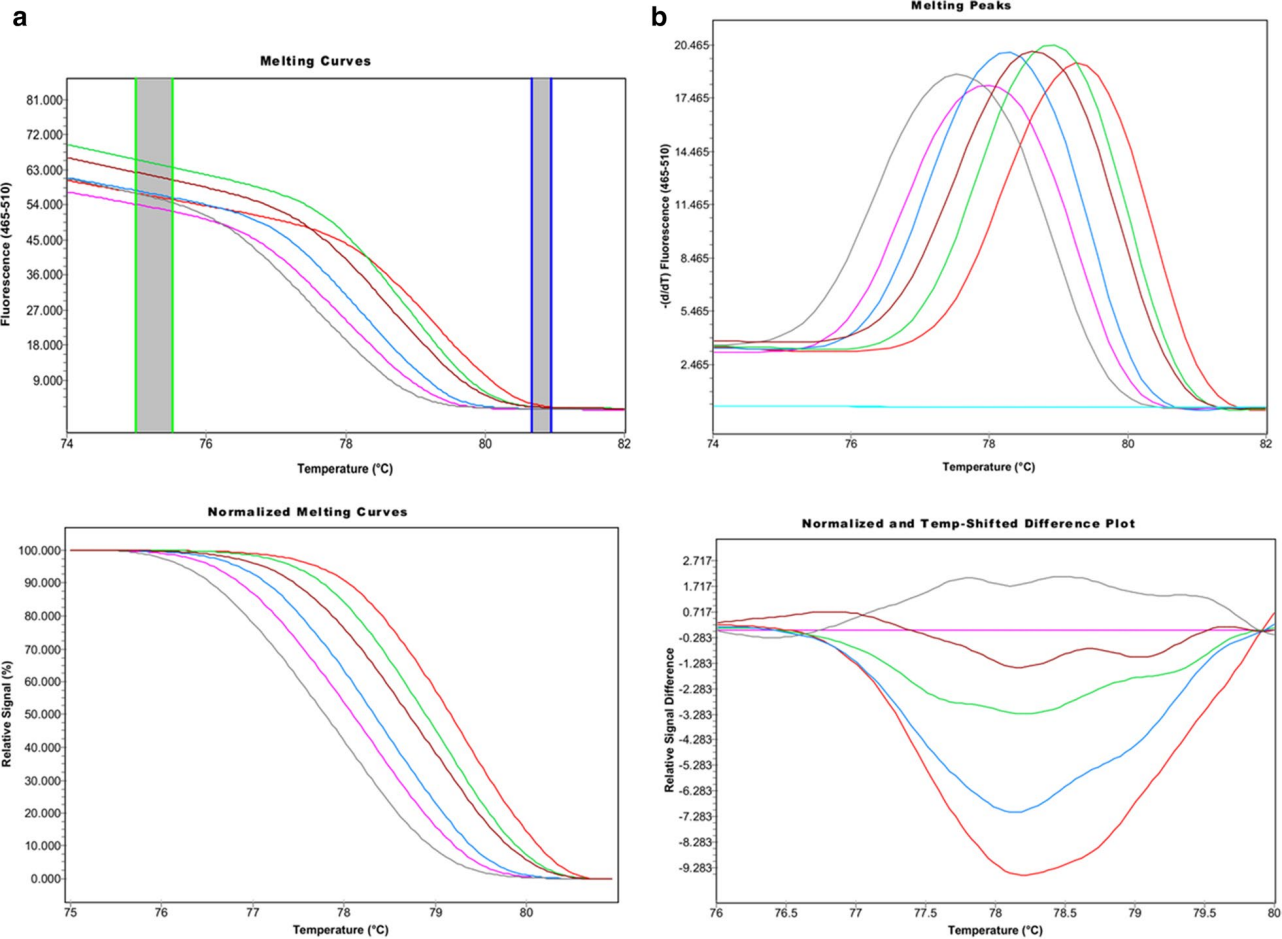
Melting curves (**a**), melting peaks (**b**), normalized melting curves, and temperature shifted difference plot of reference plasmid of *P. falciparum, P. ovale wallikeri*, *P. vivax* and *P. knowlesi*, and *P. ovale curtisi*. The following melting temperatures were observed: 77.25 °C for *P. malariae* (grey), 77.69 °C for *P. vivax* (pink), 78.11°C for *P. knowlesi* (blue), 78.53 °C for *P. ovale wallikeri* (brown), 78.73 °C for *P. ovale curtisi* (green), and 79.01 °C for *P. falciparum* (red)

**Fig. 3:**
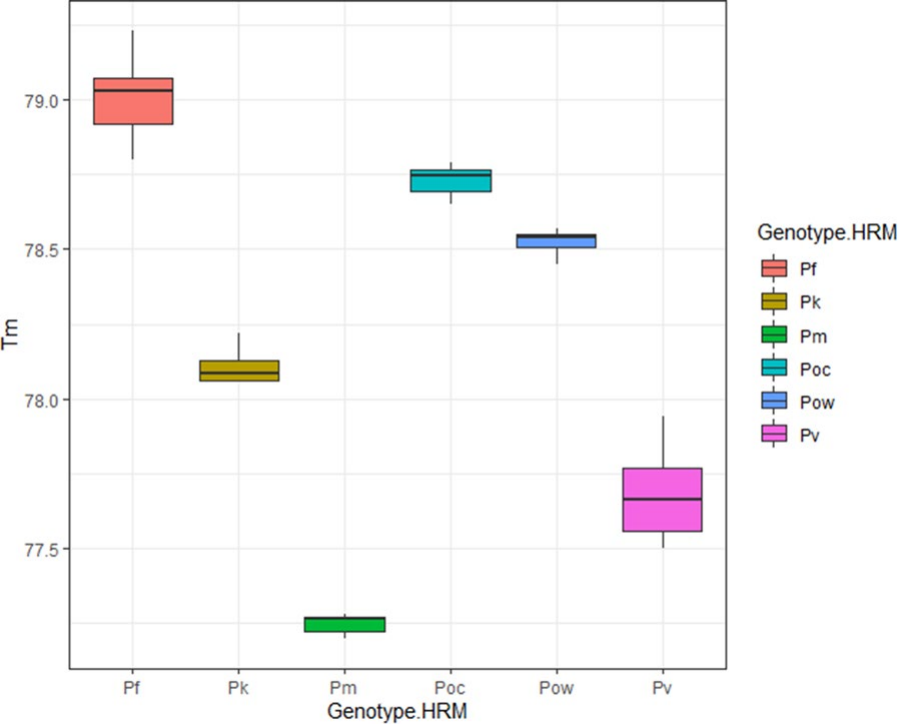
Boxplots of the melting temperature of *P. falciparum* (Pf, red), *P. ovale wallikeri* (Pow, blue), *P. vivax* (Pv, pink), *P. knowlesi* (Pk, brown), *P. malariae* (Pm, green), and *P. ovale curtisi* (Poc, teal). The box specifies the likely range of melting temperature variation. The melting temperature ranges were 77.20–77.28 °C for *P. malariae*, 77.50–77.94 °C for *P. vivax*, 78.06–78.22 °C for *P. knowlesi*, 78.45–78.57 °C for *P. ovale wallikeri*, 78.65–78.79 °C for *P. ovale curtisi*, and 78.80–79.23 °C for *P. falciparum*

Species identification was originally performed by microscopy analysis and/ or nested PCR (Additional file 1). The assay required one forward and one reverse primer binding specifically to all six human *Plasmodium* species (Fig. 1). The melting temperature (Tm) values were 77.25 ± 0.03° C (*P. malariae*), 77.69 ± 0.12° C (*P. vivax*), 78.11 ± 0.06° C (*P. knowlesi*), 78.53 ± 0.03 °C (*P. ovale wallikeri*), 78.73 ± 0.05 °C (*P. ovale curtisi*), and 79.01 ± 0.12 °C (*P. falciparum*) (Figs. 2 and 3). The high-resolution melting analysis is differentiating each *Plasmodium* species from the others with a range of ΔTm of 0.20–0.44 °C. Single infections were systematically identified based on their melting temperature described in Table 1.

**Table 1:** Assay melting temperature, specificities, and limits of detection (LOD) with confidence interval (CI)

Species	n	Test	Tm (Tm range) (°C)	Efficiency (%)	Slope	R^2^	LOD with CI (Copy/µl)
*P. malariae*	3	HRM	77.25 ± 0.03 (77.20–77.28)	99.85	− 3.3256	0.9974	21.47 (15.97–41.16)
*P. vivax*	33	HRM	77.69 ± 0.12 (77.50–77.94)	98.78	− 3.3527	0.9985	29.36 (22.61–45.85)
*P. knowlesi*	5	HRM	78.11 ± 0.06 (78.06–78.22)	99.74	− 3.3281	0.9927	42.47 (31.67–67.57)
*P. ovale wallikeri*	13	HRM	78.53 ± 0.03 (78.45–78.57)	94.99	− 3.4482	0.9992	33.47 (24.49–57.87)
*P. ovale curtisi*	5	HRM	78.73 ± 0.05 (78.65–78.79)	97.67	− 3.3791	0.9985	30.26 (22.68–49.81)
*P. falciparum*	38	HRM	79.01 ± 0.12 (78.80–79.23)	97.95	− 3.3720	0.9991	46.43 (32.50–85.44)
*P. falciparum*	38	Probe	–	97.18	− 3.3913	0.9988	–

### Assay performance

Isolated plasmid construct with each mitochondrial fragment of the *Plasmodium* species was used to determine the efficiency of the assay by amplifying 10-fold serial dilutions starting with 10^7^ copies/µl to 10 copies/µl. The ranges of efficiency, slope, and R^2^ were 94.99 to 99.85 %, − 3.3256 to − 3.4482, and 0.9927 to 0.9992, respectively (Table 1). The probit analysis of runs between 80 and 10 copies /µl provided LODs at 95 % confidence varying from 21.47 (15.97–41.16) copies /µl with *P. malariae* to 46.43 (32.50–85.44) copies /µl with *P. falciparum* (Table 1).

The discrimination power of the assay was tested using 108 *Plasmodium* positive samples, 5 *Plasmodium* negative samples, and also DNA samples positive for 16 other organisms listed in section material and methods. The melting temperature (Tm) results of the *Plasmodium* samples are illustrated in boxplots (Fig. 3). The Welch’s unequal variances t-test showed that Tm of all *Plasmodium* amplicon (6) was significantly different (p-value < 2.2e−16).

**Fig. 4:**
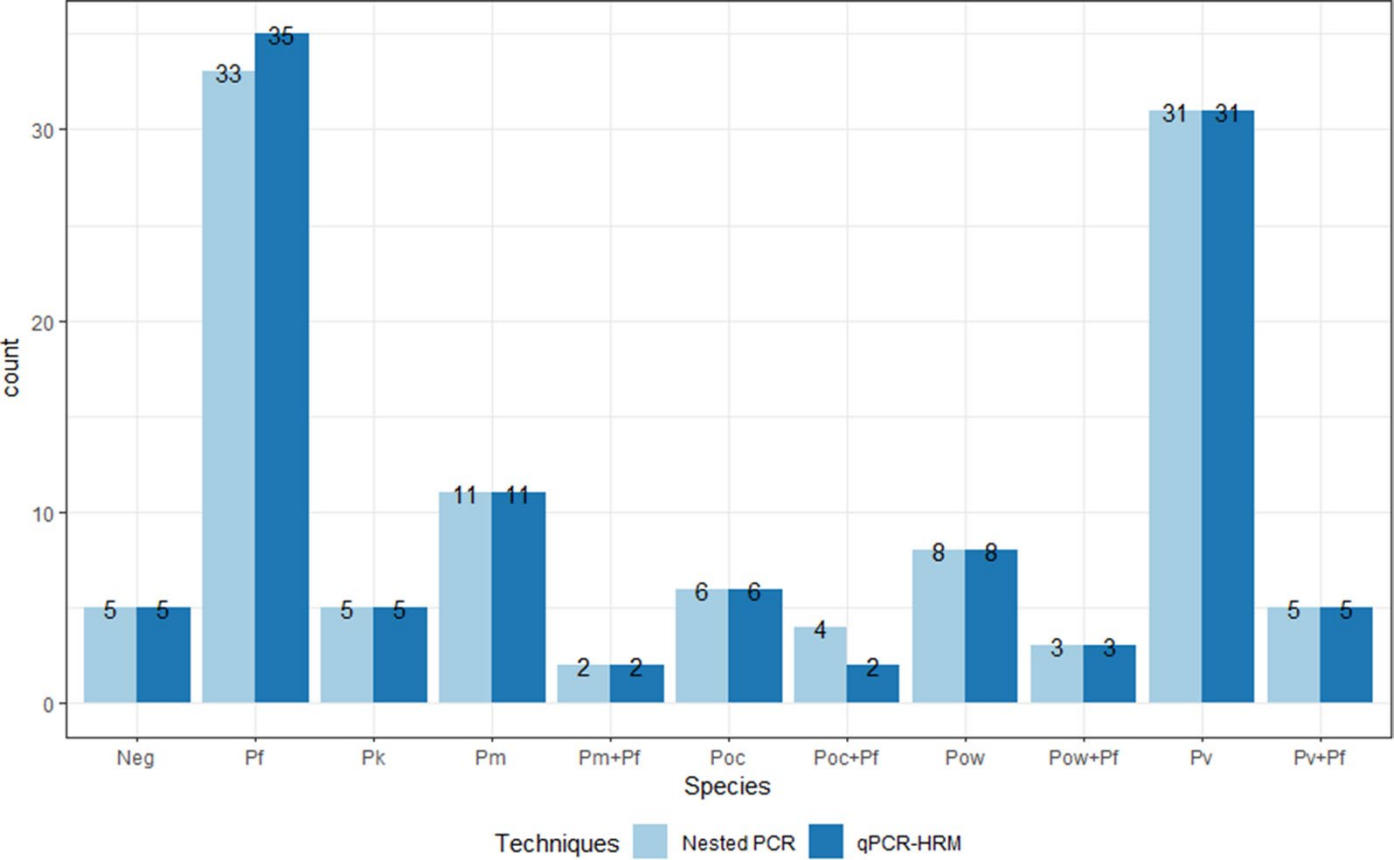
qPCR-HRM (azure blue) results of 113 samples compared to nested PCR (baby blue). The qPCR-HRM results of 113 (98 %) samples matched with the nested PCR. Two mixed infections of *P. falciparum/ P. ovale curtisi* were detected as a single infection of *P. falciparum* in the developed qPCR-HRM assay

**Fig. 5:**
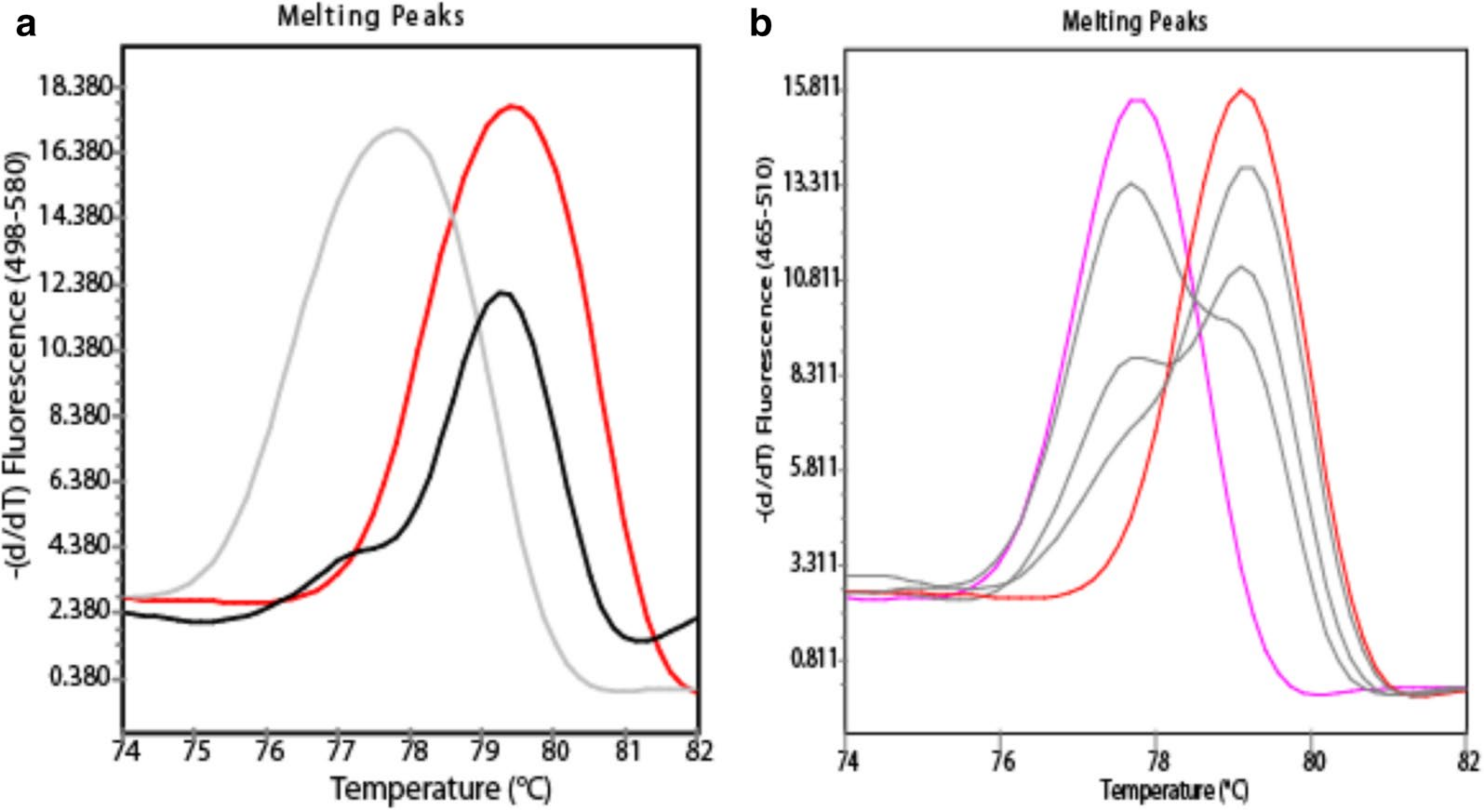
Melting peaks of mixed infection samples presenting 2 cases of double infection:** a** *P. malariae* (grey), *P. falciparum* (red) with a mixed infection *P. malariae/ P. falciparum* (black).** b** *P. vivax* (pink), *P. falciparum* (red) with a mixed infection *P. vivax/ P. falciparum* (grey)

Out of the 113 samples tested with the HRM-qPCR- *P. falciparum* probe assay, 96 (85.0 %) single infections were detected: 11 *P. malariae*, 5 *P. knowlesi*, 6 *P. ovale curtisi*, 8 *P. ovale wallikeri*, 31 *P. vivax* and 35 *P. falciparum* were detected (Fig. 4). Additionally, 12 (10.6 %) mixed infections of *P. malariae* /*P. falciparum* (2), *P. falciparum / P. ovale curtisi* (2), *P. vivax/ P. falciparum* (5), and *P. falciparum* / *P. ovale wallikeri* (3), were identified. Five (4.4 %) samples were *Plasmodium* negative. All mix infections were observed with *P. falciparum* infection confirmed by high resolution melting and/or by the probe with texas-red detection at 650nm. The HRM melting curves alone allowed the detection of two of the four mixed infections (*P. falciparum/ P. malariae*, and *P. falciparum/ P. vivax*) (Fig. 5) confirmed by the Taqman probe amplification curve. The two additional mixed infections *P. falciparum/ P. ovale curtisi*, and *P. falciparum/P. ovale wallikeri* needed both HRM Tm (for *P. ovale* species) and Taqman probe amplification (for *P. falciparum*) to be confirmed.

The results were concordant with those of the nested PCR at 98.2 % at 95 % CIs. Indeed, out of the 113 samples, two mixed infections of *P. falciparum/P. ovale curtisi* (nested PCR) were detected as single infections with *P. falciparum* using the qPCR-HRM-*P. falciparum* probe assay. The selected forward and reverse primers were specifically binding to all six *Plasmodium* species and did not amplify any of the 16 organisms used to test the specificity of the method.

## Discussion

A combination of an intercalating dye with a hydrolysis probe real-time PCR is described with a simultaneous differentiation of all human *Plasmodium* species. The hydrolysis probe designed for *P. falciparum* was included to double differentiate the predominant and potentially most virulent parasite (*P. falciparum*) from the other *Plasmodium* species: *P. ovale wallikeri, P. ovale curtisi, P. vivax, P. malariae*, and *P. knowlesi*. In an attempt to increase the assay specificity and sensitivity, the mitochondrial genome was targeted because it is more conserved within each of the *Plasmodium* species and exists in multiple copies (up to ~ 22 copies) within each parasite [13–16]. The developed assay’s specificity (98.2 % at 95 % CIs) and sensitivity (LODs of 21.47–46.43 copies/µl, equivalent to 1–2.11 parasites/µl) are comparable to those of other studies like Joste et al. [27] and Murillo et al. [28] with 100 % specificity and 1 parasite/µl sensitivity. Chua et al. [21] reported a sensitivity range of 1–100 copies/µl in a qPCR-HRM assay targeting the 18S SSU rRNA gene of *Plasmodium* spp. with also one primer set. Similar LODs values of 1parasite/µl and 1–10 parasites/µl were also achieved by Lucchi et al. [29] and Demas et al.[30] with *P. knowlesi*, and *P. falciparum*-*P. vivax*, respectively. Further studies have shown that lower LODs can be achieved when the qPCR target has higher copy number like the teleromic-associated repetitive element 2 (TARE-2, ~ 250 copies/genome) and the *var* gene acidic terminal sequence (varATS, 59 copies/genome). Indeed, lower LODs (0.03–0.15 parasites/µl) were achieved by Hofmann et al. [31] using the TARE-2 and varATS, respectively for *P. falciparum* and *P. vivax*. More recently, Gupta et al. [32] reported 34–44 copies of PfMLS152 and PvMLS110 sequences corresponding to *P. falciparum* and *P. vivax*, respectively, with a low LOD value (0.1 parasites/µl). The qPCR assay also demonstrated the utility of multi-copy DNA sequence in the diagnosis of malaria. Its sensitivity is lower compare to other studies but the developed assay is presenting the advantage to target a conserved fragment of the mitochondria genome compare to the poor homology of the repeats in assays with higher copy of genomic sequences [31, 32].

The time needed to run a sample, including both amplification curves detection and melting curve analysis, was 1h15 min without a probe, and 2 h when the Texas-red probe was added, respectively. The qPCR-HRM developed by Chua et al. [21] was also performed in 2 hours. These single PCR techniques are faster compared to the conventional approaches based on nested PCR with additional PCR product separation by electrophoresis in agarose gel [23, 33, 34].

This assay has the advantage of detecting all *Plasmodium* species with a single primer set in one PCR reaction. Additionally, *P. falciparum* infection is confirmed with the hydrolysis probe in the same PCR reaction. Mixed infections remain a major challenge even for experienced microscopists and are difficult to detect with most currently available RDTs. Such a method will therefore be essential in co-endemic areas where species differentiation is crucial for directing appropriate treatment and surveillance [17]. This assay provides a sensitive and rapid method to overcome the difficulties with distinguishing mixed infections involving *P. falciparum*, *e.g., P. knowlesi/ P. falciparum* [35], or *P. malariae/ P. falciparum* [36].

A limitation of the current assay is the identification of selected mixed infections due to the small Tm difference between the species. Indeed, mixed infections involving the two *P. ovale* species (*wallikeri* and *curtisi*) would be detected as a single infection with the developed assay. This, however, has very limited clinical implications. In this case, a method targeting the two closely related *P. ovale* species like the one previously developed [22], are necessary to differentiate the species.

The use of a Taqman probe (250 $ for ~ 6000 qPCR reactions) was generating additional cost making the developed assay slightly more expensive than the SYBR Green detection assays [21]. But the probe cost is distribution across thousands of PCR reactions making low impact on the cost of sample analysis. Despite using a Taqman probe, the developed assay remains cost-efficient compared to a fluorescence resonance energy transfer (FRET) more expensive than the Taqman probes [37–39]. The assay without the Taqman probe will indeed identify any *Plasmodium* infection through the specific melting temperature.

## Conclusions

This is the first method describing the combination of non-probe (HRM) with a hydrolysis probe qPCR in malaria diagnosis. The assay is targeting all six *Plasmodium* species with an additional detection step for *P. falciparum* infection. The assay provides a highly sensitive, specific, and easy to perform HRM-hydrolysis probe qPCR assay for differentiating and quantifying malaria parasites. This qPCR assay could contribute to a timely diagnosis in both non-malaria-endemic and malaria-endemic areas and also contribute to protecting the most vulnerable population groups, like young children and non-immune populations, in whom *P. falciparum* can be rapidly fatal.

## Supplementary Information

**Additional file 1: Table S1.** List of samples used to evaluate the developed qPCR-HRM assay with their microscopy, nested PCR, and qPCR-HRM genotyping.

## Data Availability

The datasets used and/or analysed during the current study are available from the corresponding author on reasonable request.

## Abbreviations

BLAST: Basic local alignment search tool
DNA: Deoxyribonucleic acid
FRET: Fluorescence resonance energy transfer
HRM: High resolution melting
LOD: Limit of detection
PCR: Polymerase chain reaction
Tm: Melting temperature
TARE: Teleromic-associated repetitive element 2
varATS: Var gene acidic terminal sequence
PfMLS152: *Plasmodium falciparum* multiloci short stretch of 152
PvMLS110: *Plasmodium vivax* multiloci short stretch of 110
