# Supplementary material for: A powerful qPCR-high resolution melting assay with taqman probe in plasmodium species differentiation
Source: Malar J. 2021 Feb 28;20:121. doi: 10.1186/s12936-021-03662-w (PMC7916309; doi:10.1186/s12936-021-03662-w)
Supplement: Supplementary file 1 — Additional file 1: Table S1. List of samples used to evaluate the developed qPCR-HRM assay with their microscopy, nested PCR, and qPCR-HRM genotyping. [file 12936_2021_3662_MOESM1_ESM.docx]

**Additional File 1**

Table: Samples liste

| No. | Sample ID | Origin | Microscopy^a^ | Parasite density/µl^c^ | Nested PCR | qPCR-HRM Genotyping | Probe Pf Genotyping |
| --- | --- | --- | --- | --- | --- | --- | --- |
| 1 | Po1 | Bangladesh | Pv | 2240 | Pow | Pow |  |
| 2 | Po2 | Bangladesh | Pm | 6680 | Pow | Pow |  |
| 3 | Po3 | Bangladesh | Pm | 2600 | Pow | Pow |  |
| 4 | Po4 | Bangladesh | Pv | 280 | Poc | Poc |  |
| 5 | Po5 | Bangladesh | Pv | 120 | Pow | Pow |  |
| 6 | Po7 | Bangladesh | Pv | 440 | Pow/Pf | Pow | Pf |
| 7 | Po9 | Bangladesh | Pf | 14520 | Pow/Pf | Pow | Pf |
| 8 | Po11 | Bangladesh | neg | - | Pow | Pow |  |
| 9 | Po12 | Bangladesh | neg | - | Poc | Poc | Pf |
| 10 | Po14 | Bangladesh | neg | - | Poc | Poc |  |
| 11 | Po15 | Bangladesh | neg | - | Poc | Poc |  |
| 12 | Po16 | Bangladesh | neg | - | Poc/Pf | Poc |  |
| 13 | Po18 | Bangladesh | neg | - | Poc/Pf | Poc | Pf |
| 14 | T5 | Ethiopia | Pf |  | Pf | Pf | Pf |
| 15 | T7 | Ethiopia | Pv |  | Pv | Pv |  |
| 16 | T8 | Ethiopia | Pf |  | Pf | Pf | Pf |
| 17 | T17 | Ethiopia | Pf |  | Pf | Pf | Pf |
| 18 | T32 | Ethiopia | Pv |  | Pv | Pv |  |
| 19 | T52 | Ethiopia | neg |  | Pow | Pow | Pf |
| 20 | K1 | Ethiopia | Pf |  | Pf | Pf | Pf |
| 21 | K3 | Ethiopia | Pf |  | Pf | Pf | Pf |
| 22 | K5 | Ethiopia | Pf |  | Pf | Pf | Pf |
| 23 | K7 | Ethiopia | Pf |  | Pf | Pf | Pf |
| 24 | K10 | Ethiopia | Pf |  | Pf | Pf | Pf |
| 25 | K11 | Ethiopia | Pf |  | Pv | Pf | Pf |
| 26 | K13 | Ethiopia | Pf |  | Pf | Pf | Pf |
| 27 | K17 | Ethiopia | Pf |  | Pf | Pf | Pf |
| 28 | K19 | Ethiopia | Pf |  | Pf | Pf | Pf |
| 29 | K22 | Ethiopia | Pf |  | Pv/Pf | Pv/Pf | Pf |
| 30 | K41 | Ethiopia | Pv |  | Pf/Poc | Poc | Pf |
| 31 | K21 | Ethiopia | Pv |  | Pf/Pow | Pow | Pf |
| 32 | M4 | Ethiopia | Pf |  | Pf | Pf | Pf |
| 33 | M5 | Ethiopia | Pf |  | Pf | Pf | Pf |
| 34 | M8 | Ethiopia | Pf |  | Pf | Pf | Pf |
| 35 | M10 | Ethiopia | Pf |  | Pf | Pf | Pf |
| 36 | M12 | Ethiopia | Pv |  | Pv | Pv |  |
| 37 | M16 | Ethiopia | Pv |  | Pv | Pv |  |
| 38 | M17 | Ethiopia | Pf |  | Pv | Pv |  |
| 39 | M18 | Ethiopia | Pf |  | Pf | Pf | Pf |
| 40 | M19 | Ethiopia | Pv |  | Pv | Pv |  |
| 41 | M20 | Ethiopia | Pf |  | Pf | Pf | Pf |
| 42 | M32 | Ethiopia | pv |  | Pv | Pv |  |
| 43 | M64 | Ethiopia | Pf |  | Pf | Pf | Pf |
| 44 | M66 | Ethiopia |  |  | Pv/Pf | Pv/Pf | Pf |
| 45 | F13 | Ethiopia | Pv |  | Pv | Pv |  |
| 46 | F17 | Ethiopia |  |  | Pv/Pf | Pv/Pf | Pf |
| 47 | F28 | Ethiopia | Pv |  | Pv | Pv |  |
| 48 | F32 | Ethiopia | Pv |  | Pv | Pv |  |
| 49 | F33 | Ethiopia | Pv |  | Pv | Pv |  |
| 50 | F34 | Ethiopia | Pv |  | Pv | Pv |  |
| 51 | F36 | Ethiopia | Pv |  | Pv | Pv |  |
| 52 | F38 | Ethiopia | Pv |  | Pv | Pv |  |
| 53 | F41 | Ethiopia | Pv |  | Pv | Pv |  |
| 54 | F42 | Ethiopia | Pf |  | Pv | Pf | Pf |
| 55 | F44 | Ethiopia | Pv |  | Pv | Pv |  |
| 56 | F48 | Ethiopia | Pv |  | Pv | Pv |  |
| 57 | F80 | Ethiopia | Pv |  | Pv | Pv |  |
| 58 | Pm1 | Bangladesh | - |  | Pm | Pm |  |
| 59 | Pm2 | Bangladesh | - |  | Pm | Pm |  |
| 60 | Pm3 | Bangladesh | - |  | Pm | Pm |  |
| 61 | Pk2 | Malaysia | - |  | Pk | Pk |  |
| 62 | Pk3 | Malaysia | - |  | Pk | Pk |  |
| 63 | Pk4 | Malaysia | - |  | Pk | Pk |  |
| 64 | SG1821 | Ethiopia |  | 0 | Pf/Poc | Poc | Pf |
| 65 | SG1835 | Ethiopia |  | 1760 | Pv | Pv |  |
| 66 | SG1845 | Ethiopia |  | 0 | Pv | Pv |  |
| 67 | AK 12 | Ethiopia |  | 2000 | Pf | Pf | Pf |
| 68 | AK 16 | Ethiopia |  | 1600 | Pv | Pv |  |
| 69 | AK 23 | Ethiopia |  | 4000 | Pf | Pf | Pf |
| 70 | AK 25 | Ethiopia |  | 0 |  | Negative |  |
| 71 | AK 26 | Ethiopia |  | 4000 | Pf | Pf | Pf |
| 72 | AK 60 | Ethiopia |  | 32000 | Pf | Pf | Pf |
| 73 | NB 05 | Ethiopia |  | 7800 | Pv | Pv |  |
| 74 | NB 06 | Ethiopia |  | 23400 | Pf | Pf | Pf |
| 75 | NB 10 | Ethiopia |  | 23400 | Pv | Pv |  |
| 76 | NB 14 | Ethiopia |  | 19500 | Pv | Pv |  |
| 77 | NB 22 | Ethiopia |  | 0 | Negative | Negative |  |
| 78 | NB 26 | Ethiopia |  | 15600 | Pf | Pf | Pf |
| 79 | NB 36 | Ethiopia |  | 0 | Negative | Negative |  |
| 80 | NB 39 | Ethiopia |  | 0 | Pv | Pv |  |
| 81 | NB 43 | Ethiopia |  | 15600 | Pf/Pv | Pv | Pf |
| 82 | NB 44 | Ethiopia |  | 12480 | Pv | Pv |  |
| 83 | NB 49 | Ethiopia |  | 0 | Negative | Negative |  |
| 84 | NB 56 | Ethiopia |  | 7800 | Pv | Pv |  |
| 85 | NB 60 | Ethiopia |  | 15600 | Pf | Pf | Pf |
| 86 | NB 62 | Ethiopia |  | 21840 | Pf | Pf | Pf |
| 87 | NB 69 | Ethiopia |  | 0 | Negative | Negative |  |
| 88 | NB 70 | Ethiopia |  | 12480 | Pv | Pv |  |
| 89 | NB 71 | Ethiopia |  | 9360 | Pf | Pf | Pf |
| 90 | NB 73 | Ethiopia |  | 18720 | Pf | Pf | Pf |
| 91 | 130 | Bangladesh |  |  |  | Pm |  |
| 92 | 402 | Bangladesh |  |  |  | Pm/Pf | Pf |
| 93 | 800 | Bangladesh |  |  |  | Pm |  |
| 94 | 1270 | Bangladesh |  |  |  | Pm |  |
| 95 | 1627 | Bangladesh |  |  |  | Pm/Pf | Pf |
| 96 | 2406 | Bangladesh |  |  |  | Pm |  |
| 97 | 2475 | Bangladesh |  |  |  | Pm |  |
| 98 | 2489 | Bangladesh |  |  |  | Pm |  |
| 99 | 2725 | Bangladesh |  |  |  | Pm |  |
| 100 | 2736 | Bangladesh |  |  |  | Pm |  |
| 101 | Pf1 | Ethiopia |  |  | Pf | Pf | Pf |
| 102 | Pf2 | Ethiopia |  |  | Pf | Pf | Pf |
| 103 | Pka | Malaysia |  |  | Pk | Pk |  |
| 104 | Pkb | Malaysia |  |  | Pk | Pk |  |
| 105 | Poc1 | Ethiopia |  |  | Poc | Poc |  |
| 106 | Poc2 | Ethiopia |  |  | Poc | Poc |  |
| 107 | Pow1 | Ethiopia |  |  | Pow | Pow |  |
| 108 | Pow2 | Ethiopia |  |  | Pow | Pow |  |
| 109 | Pv1 | Ethiopia |  |  | Pv | Pv |  |
| 110 | Pv2 | Ethiopia |  |  | Pv | Pv |  |
| 111 | Pv3 | Ethiopia |  |  | Pv | Pv |  |
| 112 | K34 | Ethiopia | Pf |  | Pv/Pf | Pv/Pf | Pf |
| 113 | K39 | Ethiopia | Pf |  | Pv/Pf | Pv/Pf | Pf |
